# Neurologic Complications in Heart Transplant Recipients Readmitted to the Intensive Care Unit

**DOI:** 10.7759/cureus.19425

**Published:** 2021-11-10

**Authors:** Helin Şahintürk, Beyza Meltem Yurtsever, Özgür Ersoy, Seda Kibaroğlu, Pınar Zeyneloğlu

**Affiliations:** 1 Anesthesiology and Critical Care, Başkent University Faculty of Medicine, Ankara, TUR; 2 Cardiovascular Surgery, Dışkapı Yıldırım Beyazıd Eğitim ve Araştırma Hastanesi, Ankara, TUR; 3 Neurology, Başkent University Faculty of Medicine, Ankara, TUR

**Keywords:** transplantation, icu, intensive care unit, heart transplantation, neurologic complications

## Abstract

Introduction

Neurologic complications after transplantation surgery are major causes of morbidity, and the incidence of neurologic complications among heart transplant recipients varies from 7% to 81%.

In our study, we aimed to determine the incidence, etiologies, and risk factors of neurologic complications among patients readmitted to the intensive care unit (ICU) after heart transplantation.

Method

In this retrospective cohort study, the medical records of all patients who underwent cardiac transplantation from February 2003 to July 2019 were reviewed, and those admitted to the ICU due to neurologic complications during the early and late postoperative period were evaluated. The patients were divided into two groups based on the development of neurologic complications to compare demographic and other characteristics.

Results

A total of 130 heart transplant recipients were analyzed. We excluded 33 patients from the study because they either had neurologic complications or died postoperatively without discharge from the intensive care unit. The mean age of the cohort was 35.4 ± 18.5 years, and 74 (76.3%) were male. Out of those 97 heart transplant recipients, 22 (22.7%) developed neurologic complications. Five patients (22.7% ) were admitted to the ICU in the first month, six patients (27.3%) were admitted to the ICU between one and six months, and 11 patients (50%) were admitted to the ICU six months after transplantation due to neurologic complications. The most common diagnosis was posterior reversible encephalopathy syndrome (PRES) (n = 6, 27.3%). The other diagnoses were calcineurin inhibitor toxicity (n = 5, 22.7%), intracranial hemorrhage (n = 3, 13.6%), seizures (n = 2, 9.2%), stroke (n = 2, 9.2%), femoral neuropathy (n = 1, 4.5%), myopathy (n = 1, 4.5%), phrenic nerve damage (n = 1, 4.5%), and cerebral abscess (n = 1, 4.5%). The rate of neurologic complications was higher in males when compared with females (p = 0.03). Both groups were similar in terms of the etiologies of cardiac failure, coexisting disease, and anticoagulant and immunosuppressive usage. The requirement for mechanical ventilation, renal replacement therapy, and the incidence of acute kidney injury were similar in both groups (p > 0.05). The incidence of sepsis was significantly higher in patients with neurologic complications (n = 8, 36.4%, versus n = 5, 6.7%; p < 0.001). The mean length of hospital stay was significantly higher in patients with neurologic complications (21.4 ± 15.8 versus 11.1 ± 13.3 days, p = 0.01).

The risk of developing neurologic complications is 3.036 times higher in males, and this is statistically significant (odds ratio (OR), 3.036; 95% confidence interval (CI), 1.078-8.444; p = 0.036).

Conclusion

Our results suggest that neurologic complications develop in 22.7% of heart transplant recipients admitted to the ICU, and half of them are seen after six months postoperatively. PRES was the most frequent (27.3%) neurologic complication. The risk of neurologic complications is three times higher for males. The mean length of hospital stay and incidence of sepsis were significantly higher in heart transplant recipients who developed neurologic complications.

## Introduction

Heart failure, which affects millions of people worldwide, should be considered a global health priority [1,2]. Heart transplantation is the main treatment method in patients with end-stage heart failure in whom all treatment modalities are insufficient [3]. The incidence of neurologic complications after heart transplantation varies between 7% and 81% [4-6]. Cerebrovascular complications such as ischemic stroke or transient ischemic attack are usually observed. There are reports that the most common neurologic complication after cardiac transplantation is posterior reversible encephalopathy syndrome (PRES) and develops secondary to immunosuppressive therapy [6].

Heart transplantation has been performed in our clinic since 2003. Recently, the rate of heart transplantation has increased in our country and in the world. Our clinical observation is that patients have a high incidence of neurologic complications after heart transplantation.

Although there are publications about neurologic complications after heart transplantation, we aimed to determine the neurologic complications, incidence, and risk factors among patients readmitted to the intensive care unit (ICU) after heart transplantation.

## Materials and methods

This study was approved by Başkent University Institutional Review Board (KA 19/205) and supported by Başkent University Research Fund. In this retrospective cohort study, the medical records of all patients who underwent cardiac transplantation from February 2003 to July 2019 were reviewed. Among those, patients admitted to the ICU due to neurologic complications during the early and late postoperative period were evaluated. Patients who developed neurologic complications before discharge from the intensive care unit after surgery and who died in the early postoperative period without discharge from the intensive care unit were excluded from the study. The patients were divided into two groups based on the development of neurologic complications to compare demographic and other characteristics. The collected data included the demographic characteristics of patients (age, gender, body weight, and body height), comorbidities, drugs, perioperative laboratory values and hemodynamic parameters, intraoperative features, length of mechanical ventilation, lengths of ICU/hospital stay, and hospital mortality.

The same opioid-based anesthesia technique was used in all heart transplant recipients. Anesthesia was induced with midazolam 0.02-0.05 mg/kg intravenous (i.v.), fentanyl 500 μg i.v., and rocuronium bromide 0.6-1 mg/kg i.v. Desflurane at a concentration of 4%-6% and fentanyl 5 μg/kg/h were used for the maintenance of anesthesia. In all cases, our clinical protocols for standard cardiopulmonary bypass (CPB) surgery and standard perfusion techniques were used. Carbon dioxide insufflation within the surgical field was used to prevent microemboli. Following surgery, all patients were admitted to the intensive care unit intubated and mechanically ventilated. The same surgical, anesthesia, and intensivist teams were assigned during the perioperative period for all heart transplantation surgeries.

The patients who developed neurologic complications were divided into three groups according to the time of development: those who developed neurologic complications in the first month, between one and six months, and after six months. Neurologic complications were evaluated as early postoperative complications in the first month and late postoperative complications after six months.

Data were summarized as mean ± standard deviation and median (range) for continuous variables and frequencies (percentiles) for categorical variables. Student’s t-test or Mann-Whitney U test was used for independent group comparisons, depending on the distributional properties of the data. The Chi-square test was used for proportions, and its counterpart, Fisher’s exact test, was used when the data were sparse. To define risk factors for the outcome variable (neurologic complications), multiple logistic regression analysis was used, and adjusted odds ratios (ORs) and their confidence intervals (CIs) were calculated. All analyses were performed using IBM SPSS Statistics for Windows, version 20.0. A p value < 0.05 was considered statistically significant.

## Results

A total of 130 heart transplant recipients were analyzed. We excluded 33 patients from the study because they either had neurologic complications postoperatively without discharge from the intensive care unit or they died postoperatively without discharge from the intensive care unit. The mean age of the cohort was 35.4 ± 18.5 years, and 74 (76.3%) were male. The demographic characteristics and main diagnosis of the patients included in the study are shown in Table 1.

**Table 1:** Demographic characteristics and main diagnosis of the study population [mean ± SD or n (%)]

	n = 97 (%)
Age (years)	35.4 ± 18.5
Male	74 (76.3%)
Diagnosis	
Dilated cardiomyopathy	59 (60.8%)
Restrictive cardiomyopathy	12 (12.4%)
Ischemic cardiomyopathy	15 (15.5%)
Myocarditis	3 (3.1%)
Congenital heart disease	7 (7.2%)
Arrhythmogenic right ventricular cardiomyopathy	1 (1%)

Out of those 97 heart transplant recipients, 22 (23.7%) developed neurologic complications. Five patients (22.7% ) were admitted to the ICU in the first month, six patients (27.3%) were admitted to the ICU between one and six months, and 11 patients (50%) were admitted to the ICU six months after transplantation due to neurologic complications (Table 2).

**Table 2:** Timing of neurologic complications

Postoperative	n (22)	% (100%)
One month	5	22.7
One to six months	6	27.3
>6 months	11	50

The most common diagnosis was PRES (n = 6, 27.3%). The other diagnoses were calcineurin inhibitor toxicity (n = 5, 22.7%), intracranial hemorrhage (n = 3, 13.6%), seizures (n = 2, 9.2%), stroke (n = 2, 9.2%), femoral neuropathy (n = 1, 4.5%), myopathy (n = 1, 4.5%), phrenic nerve damage (n = 1, 4.5%), and cerebral abscess (n = 1, 4.5%) (Table 3).

**Table 3:** Neurologic complications PRES: posterior reversible encephalopathy syndrome

	n (22)	% (100%)
PRES	6	27.3
Calcineurin inhibitor toxicity	5	22.7
Hemorrhage	3	13.6
Seizures	2	9.2
Stroke	2	9.2
Femoral neuropathy	1	4.5
Phrenic nerve damage	1	4.5
Myopathy	1	4.5
Brain abscess	1	4.5

The rate of neurologic complications was higher in males than in females (p = 0.03). There was no difference in the frequency of neurologic complications between patients ≤18 years and >18 years (p = 0.754). Both groups were similar in terms of the etiologies of cardiac failure, coexisting disease, and anticoagulant and immunosuppressive usage (Table 4).

**Table 4:** Comparison of the two groups in terms of demographics, etiologies, coexisting disease, and immunosuppressive drugs [mean ± SD or n (%)] *p value of Fisher’s exact test **p value < 0.05 NC: neurologic complications, ICMP: ischemic cardiomyopathy, DCMP: dilated cardiomyopathy, RCMP: restrictive cardiomyopathy, ARVC: arrhythmogenic right ventricular cardiomyopathy, HTN: hypertension, DM: diabetes mellitus

		NC (-)	NC (+)	p
Age	≤18	18 (24)	6 (27.3)	0.754
	>18	57 (76)	16 (72.7)	
Gender	Male	61 (81.3)	13 (59.1)	0.031**
	Female	14 (18.7)	9 (40.9)	
Etiologies	ICMP	14 (18.7)	1 (4.5)	0.086*
	DCMP	40 (53.3)	19 (86.5)	
	RCMP	11 (14.7)	1 (4.5)	
	Myocarditis	2 (2.7)	1 (4.5)	
	Congenital	7 (9.3)	0 (0)	
	ARVC	1 (1.3)	0 (0)	
Coexisting disease	HTN	4 (5.3 )	2 (9.1)	0.222*
	Pulmonary HTN	5 (6.7)	1 (4.5)	
	DM	1 (1.3)	1 (4.5)	
	Cardiac failure	55 (73.5)	12 (54.5)	
	Chronic renal failure	1 (1.3)	0 (0)	
	Malignancy	1 (1.3)	1 (4.5)	
	Arrhythmia	4 (5.3)	1 (4.5)	
	Others	4 (5.3)	4 (18.2)	
Immunosuppressive agent	Tacrolimus	59 (78.7)	17 (77.3)	0.788*
	Cyrolimus	13 (17.3)	5 (22.7)	
	Cyclosporine	3 (4)	0 (0)	

The requirement for mechanical ventilation, renal replacement therapy, and the incidence of acute kidney injury were similar in the two groups (p > 0.05) (Table 5). The incidence of sepsis was significantly higher in patients with neurologic complications (n = 8, 36.4%, versus n = 5, 6.7%; p < 0.001). The mean length of hospital stay was significantly higher in patients with neurologic complications (21.4 ± 15.8 versus 11.1 ± 13.3 days, p = 0.01), but the mean length of ICU stay was similar in both groups (8.8 ± 8.7 versus 9.1 ± 12.3 days, p = 0.9). Hospital mortality (50% versus 44%, p = 0.6) and 30-day mortality (27.7% versus 41.3%, p = 0.1) were not different in the two groups (Table 5).

**Table 5:** Comparison between the two groups in terms of outcomes [mean ± SD or n (%)] *p value of Fisher’s exact test **p value < 0.05 NC: neurologic complications, AKI: acute kidney injury, RRT: renal replacement therapy, MV: mechanical ventilation, ICU: intensive care unit

	NC (-)	NC (+)	p
Sepsis	5 (6.7%)	8 (36.4%)	<0.001**
AKI	19 (25.3%)	4 (18.2%)	0.488
RRT requirement	17 (22.7%)	3 (13.6%)	0.357
Need for intubation	33 (44%)	10 (45.5%)	0.904
Need for MV	34 (45.3%)	11 (50%)	0.700
Duration of MV (hours)	7 ± 10.24	4.90 ± 5.26	0.714
Tracheostomy	7 (9.3%)	1 (4.5%)	0.473
Organ deficiency	20 (27%)	7 (31.8%)	0.661
Use of vasopressors	30 (40%)	8 (36.4%)	0.759
Neurologic deficit	0 (0%)	1 (4.5%)	0.227*
Length of ICU stay (days)	9.08 ± 12.28	8.76 ± 8.72	0.970
Length of hospital stay (days)	11.08 ± 13.25	21.40 ± 15.77	0.015**
Mortality (30 days)	31 (41.3%)	5 (27.7%)	0.112
ICU mortality	33 (44%)	7 (31.8%)	0.307
Hospital mortality			

We argue that using a univariate logistic regression model, gender-differentiated sample groups show statistically significant differences in the development of neurologic complications. We found that the risk of developing neurologic complications is 3.036 times higher in males, and this is statistically significant (odds ratio (OR), 3.036; 95% confidence interval (CI), 1.078-8.444; p = 0.036) (Table 6).

**Table 6:** Univariate logistic regression results **p value < 0.05 B: regression coefficient, Exp (B): odds ratio, S. error: standard error of regression coefficient (B), Min: minimum, Max: maximum, CI: confidence interval

Variables	B	S. error	Exp (B)	95% CI		p
				Min	Max	
Gender (male)	1.104	0.525	3.036	1.078	8.444	0.036**

## Discussion

In this retrospective review of 97 heart transplant recipients, the incidence of neurologic complications was estimated to be 23.7%, and 50% of them developed after six months after transplantation. The most common diagnosis was PRES. The incidence of sepsis was significantly higher in patients with neurologic complications. Being male increased the risk of neurologic complications three times. It was also established that the mean length of hospital stay was significantly higher in patients with neurologic complications.

In our study, the incidence of neurologic complications after heart transplantation was found to be 23.7%. In the literature, neurologic deficit incidence after heart transplantation varies from 7% to 81% [7-9]. We believe that the reason for this is the difference in the duration of the studies. van de Beek et al. showed that the risk of neurologic complications increased up to 81% in 15 years following heart transplantation [9]. In our study, it was found that half of the emergent neurologic complications developed after six months. In line with the findings of van de Beek et al., we think that the risk of complication development increases with time. In the literature, complications such as ischemic stroke, hemorrhagic stroke, and transient ischemic attack were mostly reviewed. Therefore, we also analyzed the incidences of seizure, calcineurin toxicity, encephalopathy, neuropathy, myopathy, and phrenic nerve damage. Thus, the reason for a higher incidence of neurologic complications in our cohort compared with other studies might be this approach.

There are publications reporting that the most frequent neurologic complication after cardiac transplantation is ischemic stroke, as well as publications reporting that the most common complication is encephalopathy [6,7,10]. The most common cause of encephalopathy has been reported as PRES [6,10]. In our study, the most common neurologic complication after heart transplantation was found to be PRES. PRES is a clinicoradiological syndrome that includes symptoms such as headache, seizure, changes in consciousness, and visual impairment [11]. Calcineurin inhibitors can have neurologic side effects even at normal serum levels without toxicity, and PRES is one of these neurologic side effects [12]. In addition to immunosuppressive therapy, renal failure and hypertension can lead to PRES development.

In our study, it has been shown that the frequency of neurologic complications is three times higher in male patients. Publications reveal that cardiac failure and morbidity after cardiac surgery is more frequent among women [2,13,14]. However, some publications advocate that there is no gender difference in the development of complications after cardiac surgery, and there are also publications reporting that neurologic complications are more frequent in male patients, similar to our study [15-18]. In their studies, Hogue et al. reported that neurologic complications were more common in women than in men, in which they investigated the effects of gender differences in neurologic outcomes and mortality after cardiac surgery, and that this was because female patients were older than male patients and had a higher rate of underlying diseases [19]. Woods et al. investigated the effect of gender on patients' outcomes after coronary artery bypass grafting and reported that the mortality and morbidity rates in women were higher than in men. They reported that the reason for this was that women had more comorbidities than men; however, they found that only neurologic complications were more common in male patients [17]. In our study, unlike Hogue et al., we found that male patients developed more neurologic complications. We attribute this to the fact that in our study population, there was no difference in men and women in terms of age and concomitant diseases.

Neurologic complications developing after heart transplantation have effects on morbidity and mortality [10]. In our study, the fact that the hospitalization duration of cardiac transplant recipients who developed neurologic complications was statistically significantly longer than that of the group without neurologic complications also coincides with this information. Additionally, the frequency of sepsis was found to be higher in the patient group with neurologic complications compared with the group without complications. We think that the reason for this is related to prolonged hospitalization. Prolonged hospitalization increases the incidence of sepsis, and sepsis increases the duration of hospitalization as well [20,21].

Limitations of this study include its retrospective, single-center nature and limited size. Future prospective studies should include larger patient groups.

## Conclusions

Our results suggest that neurologic complications develop in 22.7% of heart transplant recipients admitted to the ICU and that half of them are seen after six month postoperatively. PRES was the most frequent (27.3%) neurologic complication. The risk of neurologic complications is three times higher in the male population. The mean length of hospital stay and incidence of sepsis were significantly higher in heart transplant recipients who developed neurologic complications.
